# Prediction of the Ibuprofen Loading Capacity of MOFs by Machine Learning

**DOI:** 10.3390/bioengineering9100517

**Published:** 2022-09-30

**Authors:** Xujie Liu, Yang Wang, Jiongpeng Yuan, Xiaojing Li, Siwei Wu, Ying Bao, Zhenzhen Feng, Feilong Ou, Yan He

**Affiliations:** School of Biomedical and Pharmaceutical Sciences, Guangdong University of Technology, Guangzhou 510006, China

**Keywords:** MOFs, ibuprofen loading capacity, properties prediction, machine learning, CatBoost algorithm

## Abstract

Metal-organic frameworks (MOFs) have been widely researched as drug delivery systems due to their intrinsic porous structures. Herein, machine learning (ML) technologies were applied for the screening of MOFs with high drug loading capacity. To achieve this, first, a comprehensive dataset was gathered, including 40 data points from more than 100 different publications. The organic linkers, metal ions, and the functional groups, as well as the surface area and the pore volume of the investigated MOFs, were chosen as the model’s inputs, and the output was the ibuprofen (IBU) loading capacity. Thereafter, various advanced and powerful machine learning algorithms, such as support vector regression (SVR), random forest (RF), adaptive boosting (AdaBoost), and categorical boosting (CatBoost), were employed to predict the ibuprofen loading capacity of MOFs. The coefficient of determination (R^2^) of 0.70, 0.72, 0.66, and 0.76 were obtained for the SVR, RF, AdaBoost, and CatBoost approaches, respectively. Among all the algorithms, CatBoost was the most reliable, exhibiting superior performance regarding the sparse matrices and categorical features. Shapley additive explanations (SHAP) analysis was employed to explore the impact of the eigenvalues of the model’s outputs. Our initial results indicate that this methodology is a well generalized, straightforward, and cost-effective method that can be applied not only for the prediction of IBU loading capacity, but also in many other biomaterials projects.

## 1. Introduction

**Figure 1:**
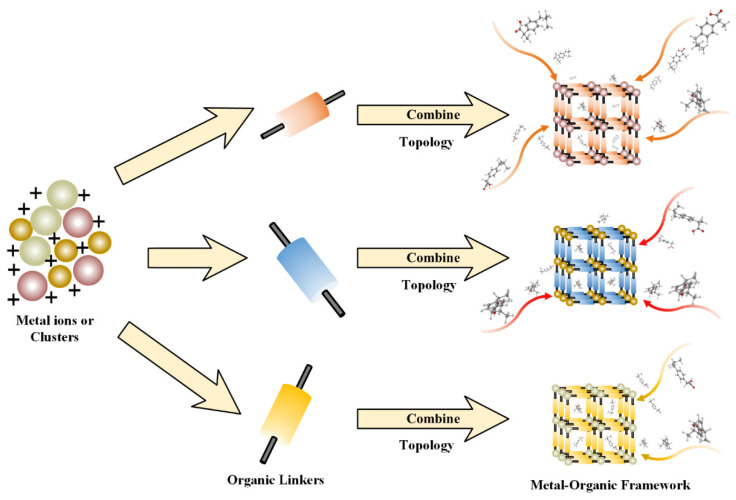
The schematic diagram of metal-organic frameworks (MOFs). The combination of various available clusters or inorganic metal ions (Fe, Cr, Zn, and so on) and organic linkers (1,4-Benzenedicarboxylate, Benzene-1,3,5-tricarboxylate, gamma−Cyclodextrin (γ-CD), and so on), which are suitable with a framework topology, can contribute to differently designed porous MOFs.

Research on metal-organic frameworks (MOFs) has drawn widespread attention, as evidenced by the significant increase in related publications [1]. MOFs are a novel class of hybrid functional materials, self-assembled from various organic linkers and metal ions/clusters [2,3] (as shown in Figure 1). MOFs usually possess desirable physicochemical properties, such as porous structures, stability, low toxicity, and modification possibilities. Hence, their popularizing applications include the storage of materials [4,5], gas separation [6,7], sensing [8], catalysis [9,10], purification [11], bio-imaging [12], and drug delivery [13,14,15]. Since MIL-101 was explored for the first time in 2006 by Ferey et al., MOFs have exhibited tremendous potentials as drug delivery systems [16].

The extraordinary properties and characteristics of MOFs mentioned above give them a significant role in drug delivery. Their strengths are apparent: firstly, their versatile structures endow them with multiple functionalities and stimuli-responsive drug-controlled release [17,18]. Secondly, their weak coordination bonds result in the biodegradability of MOFs [19]. Thirdly, the large specific surface areas and high porosity are beneficial for high loading capacity [19,20]. Fourthly, and most importantly, is that MOFs can be used as drug nano-vehicles for the treatment of various diseases, including cancer [21,22].

The number of new MOFs in private and public databases is growing exponentially [23], and conducting experiments is the most time-consuming and costly process in investigating the drug loading capacity of MOFs [24,25]. Furthermore, the achieved efficiency in batch-wise studies of MOFs’ drug loading capacity using artificial synthesis cannot be scaled up for industrial applications [25]. Thus, increasingly more efficient ways have been established to predict the drug loading capacity of MOFs. Notably, machine learning (ML) has been developed to solve these knotty problems. Compared with traditional methods, ML can decrease the calculation time significantly by utilizing the cloud disk workstations and servers [26]. ML has been employed to predict the methane adsorption capacity [27,28,29], water stability [30], toxicity [31,32], and hydrogen storage ability [33,34,35] of MOFs. To the best of our knowledge, no study has been conducted in which ML methods are employed to predict the drug loading capacity of MOFs. As a drug model, ibuprofen has been widely employed in the research regarding drug delivery systems. Furthermore, the data on ibuprofen loading capacity accounts for the majority of the data on the drug loading capacity of MOFs. In our study, we aimed to predict the drug loading capacity of MOFs using machine learning. Ibuprofen has been chosen as a model drug. We believe that ML can also be employed to predict the anticancer drug loading capacity of nanocarriers, with an adequate dataset.

Herein, the main contributions and novelty of the investigation consisted in employing the integration algorithm to predict the nonlinear IBU loading capacity of MOFs. First, we gathered 40 data points, which included the organic linkers, metal ions, and the functional groups, as well as the surface area, pore volume, and IBU loading capacity of different MOFs from more than 100 different publications. Then, we developed different powerful models for predicting IBU loading capacity, such as categorical boosting (CatBoost) [36], support vector regression (SVR) [37], random forest (RF) [38], and adaptive boosting (AdaBoost) [39]. Internally, these supervised learning methods are robust to outliers, have a low risk of overfitting, and are straightforward to use [17]. Finally, we employed the CatBoost algorithm to predict the IBU loading capacity of MOFs by means of a comprehensive assessment among all the methods. In parallel, it has a better performance estimation of the good R Squared (R^2^) and the root mean square error (RMSE) than other models and more conveniently regulates the most distinguished parameters. Furthermore, the importance of the feature effects was analyzed in terms of IBU loading capacity using the Shapley additive explanations (SHAP). This revealed that some features can influence prospective targets.

**Figure 2:**
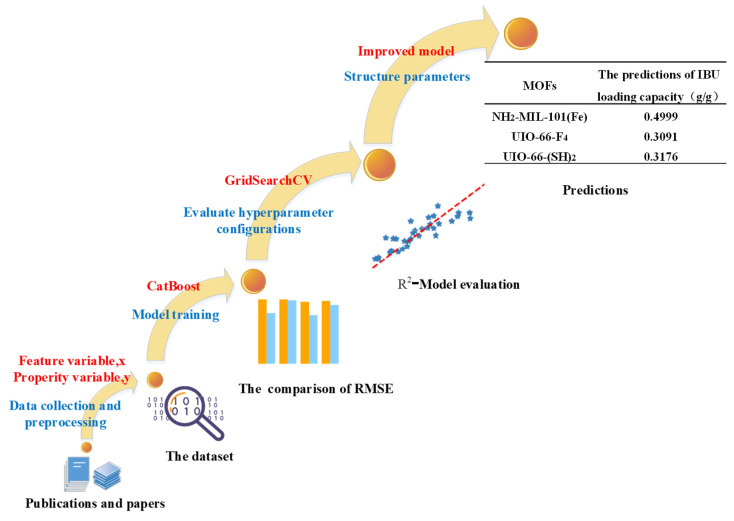
The flow chart of the research approach for the prediction of the IBU loading capacity of MOFs.

Figure 2 shows the flow chart and investigational approach of IBU loading capacity analyses. The testing dataset (hold-out data) is the input to the model, and the RMSE and R^2^ are used for the performance assessment of the model.

## 2. Materials and Methods

### 2.1. Data Acquisition

Relevant publications were thoroughly searched using authoritative institutions such as the Web of Science, Google Scholar, PubMed, Scopus, and others. We screened and extracted literature regarding MOF ibuprofen loading capacity studies from these search sites. The keywords for our literature search were: “metal organic framework”, and “drug loading”. Then, more than 100 records were retrieved from all the different search sites and narrowed down to 40 documents involving metal-centered ions, atomic clusters, organic material-linked ligands, functional groups, and IBU loading capacity of different MOFs. A matrix was used in which a row represented the category of MOFs, and the column represented the structural feature (as shown in Table 1; the complete dataset can be found in Supplementary Materials).

### 2.2. Data Processing

The collected data should be preprocessed before being input into the model to minimize the errors in model prediction, including missing value patching [46,47], feature scaling/selection [27,48], and discretization [33,49]. More specifically, to reduce the effect of the smallest and largest values on the model (for instance, the code ‘sur-area’ covers a range of surface area from 51.78 to 5510 m^2^/g), these values should be normalized [48,50] according to Equation (1).

(1) $$x^{\prime }=\frac{x-x_{min}}{x_{max}-x_{min}}$$

where x is the original eigenvalue or the encoded assigned value, and $x_{max}$ and $x_{min}$ are the maximum and minimum values in the eigenvalue dataset [27,49].

The data was preprocessed to deliver the eigenvector corresponding to the MOFs, which constituted the dataset for the model computation. The encoding was used in terms of the variables/features, indicating their presence or absence in a specific MOF. At the same time, this operation can convert the variables into a binary form that is quickly recognized by machine learning algorithms [33,36], and it can avoid a large number of decimals leading to a lengthy computation. In addition, the missing data must be filled and normalized for the next training model when analyzing the pore volume and specific surface area information. In this study, the interpolation polynomial was used to complement the missing data of pore volume and specific surface area with the original data regarding IBU loading capacity to create the new dataset. Then, the dataset was coded and input into the algorithm. The dataset was split into training and testing datasets using an allocation ratio of 0.8 and 0.2. It should be noted that the testing dataset was not used for model training, but rather as a final model verification.

### 2.3. Methodology

The CatBoost algorithm is Categorical Features+Gradient Boosting [51], based on the GBDT algorithm. It is an improved gradient boosting decision tree algorithm and an open-source and modern gradient boosting library [49,52]. It uses multiple weak learners, which are then combined into an assembled algorithm of solid learners [53]. Furthermore, it cannot only deal with intrinsically heterogeneous problems, but it can also handle categorical features [47]. This method avoids the dependence on data sorting, and is known as greedy target-based statistics, abbreviated as Greedy TS [51], and it is formulated as Equation (2).

(2) $${\hat{x}}_{k}^{i}=\frac{\sum_{j=1}^{n}I_{\left\{x_{j}^{i}=x_{k}^{i}\right\}}.y_{j}}{\sum_{j=1}^{n}I_{\left\{x_{j}=x_{k}^{i}\right\}}}$$

where I is the indicator function and $x_{k}^{i}$ is the *i*-th subtype features of the *k*-th training sample.

To reduce the differences in data structure and distribution between the training and testing dataset for feature averages and to reduce conditional bias, this algorithm randomly sorts all samples and then takes the values of particular categorical features. The preferred features and the priority weight coefficients are added as prior distribution terms [47,54]. The CatBoost algorithm uses a Greedy TS to consider combinations. It utilizes a relatively novel method of computing leaf node values in such a way (oblivious trees, symmetric trees) that it avoids the problem of overfitting that can occur with direct computation in multiple dataset arrangements [25]. The improved Greedy TS is shown in Equation (3), which reduces the effect of noisy and low-frequency category data on the data distribution [55].

(3) $${\hat{x}}_{k}^{i}=\frac{\sum_{j=1}^{n}I_{\left\{x_{j}=x_{k}^{i}\right\}}.y_{j}+\beta \cdot p}{\sum_{j=1}^{n}I_{\left\{x_{j}=x_{k}^{i}\right\}}+\beta }$$

where p is the added prior, and β is the weight of the primary, and its value is usually a coefficient greater than 0 [36]. Adding the initial probability term in the equation is a common practice for the small number of feature classes, reducing noisy data, and in the regression analysis, the initial term can be taken as the average of the dataset labels [54].

Assessing the algorithm requires quantifying the prediction errors, and it is critical to observe its uncertainty in a practical versus theoretical context. Many evaluation metrics exist for machine learning to quantify the magnitude of errors in the predictions of experimental data and intelligent models [54]. According to the algorithm, R Squared (R^2^), root mean squared error (RMSE), and mean absolute error (MAE) are the most trusted criteria. This time, R^2^ and RMSE are set as the evaluation metrics of the performance of IBU loading capacity; the smallest RMSE and largest R^2^ indicate better prediction performance of the model.

Equations (4) and (5) define the mathematical formulation of these measurement criteria [25]. They determine the mechanisms of errors in the predictive correlation of MOFs’ attributes based on machine learning and estimated IBU loading capacity performance.

(4) $$R^{2}=1-\frac{\sum_{j=1}^{N}{\left(x^{exp}-x^{cal}\right)}_{j}^{2}}{\sum_{j=1}^{N}{\left(x^{exp}-\bar{x^{cal}}\right)}_{j}^{2}}$$

The R^2^ determines the model prediction accuracy results. $x^{exp}$ is the experimental value, and $x^{cal}$ is the actual predicted value. As the predicted target gets closer to the experimental value, the R^2^ tends to be close to 1, indicating the better performance of the model prediction. Meanwhile, the RMSE statistically analyzes the error dispersion of the predictions.

(5) $$RMSE={\left(\frac{1}{N}\sum_{j=1}^{N}{\left(x^{exp}-x^{cal}\right)}_{j}^{2}\right)}^{0.5}$$

### 2.4. Computational Modeling

We utilized the learner model trees and iterations parameters with the smallest RMSE as the hyperparameters used in the model by comparing the RMSE of the training and testing dataset. Then, the R^2^ was obtained after the testing dataset as input into the model. This was conducted to evaluate the accuracy of the algorithm, which was used for the validation process. Lastly, the RMSE and R^2^ were compared for each testing dataset, and the correlative hyperparameters were used in the model. 

**Figure 3:**
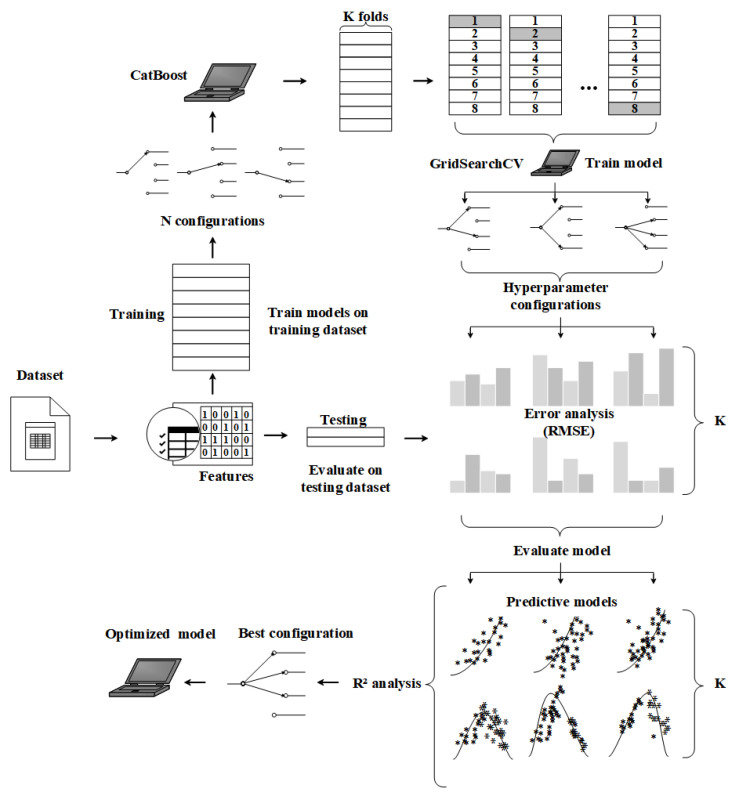
The schematic figure of the analysis pipeline showing the integrated algorithmic regression and evaluation mechanism.

The approach to the model evaluation and operation is illustrated in Figure 3. The chart determines a series of combinations of tuning hyperparameters, called N configurations, based on the type and size of the data. Hyperparameters are described as adjusting sliders. The dataset is partitioned into K-folds, and the model is trained for each fold and configuration with cross-validation (CV). Each configuration’s average performance is judged by means of the testing dataset. The preferred model is generated on the entire dataset based on the best configuration. Using the grid search, the implementation and evaluation of the best configuration are optimistic, and the optimal parameters are preserved as much as possible [46].

## 3. Results and Discussion

### 3.1. The Screening of Correlation Parameters

The applicability of the model is optimized to deliver as much important information as possible from the limited dataset. The error can be further reduced by comparing the goodness-of-fit of the training and testing scores. Then different combinations of parameters are set using K-fold cross-validation, including the number of decision trees, the learning rate, and the iterations. For instance, when the learning rate is a constant, it can match and tune different decision trees and different iterations separately. The various iterations and decision trees, combined with the grid search, are cycled to compare the RMSE until the smallest error corresponding to the parameter combinations is obtained. Finally, these parameter combinations are utilized as the model deterministic parameter of callback.

### 3.2. The Comparison of Different Machine Learning Algorithms

**Figure 4:**
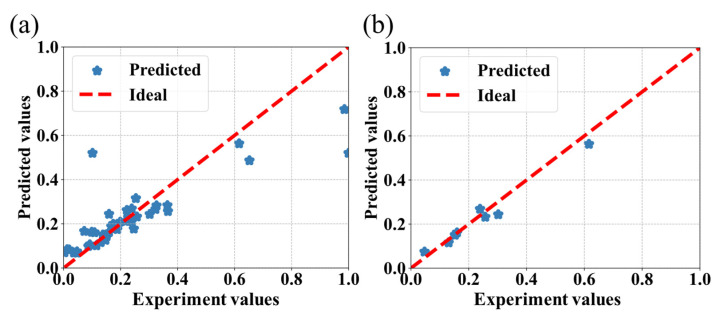
Performance of the CatBoost algorithm for the prediction of IBU loading capacity of MOFs associated with the experimental values. The values were predicted by the model, (**a**) with the entire dataset, and (**b**) with the testing dataset.

**Figure 5:**
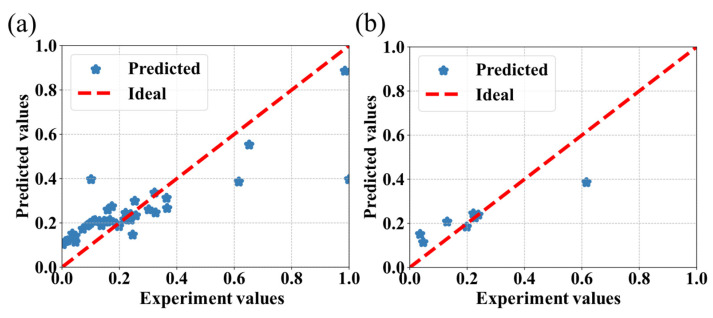
Performance of the SVR algorithm for the prediction of the IBU loading capacity of MOFs associated with the experimental values. (**a**) The predictions are calculated on the entire dataset. (**b**) The predictions are exclusively calculated on the testing dataset.

**Figure 6:**
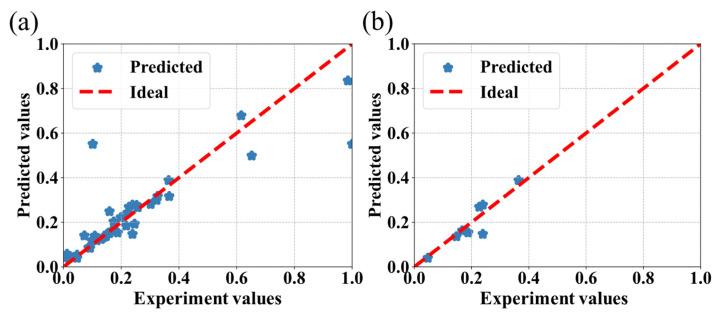
Performance of the RF algorithm for the prediction of the IBU loading capacity of MOFs associated with the experimental values. (**a**) The predictions are calculated on the entire dataset. (**b**) The predictions are exclusively calculated on the testing dataset.

**Figure 7:**
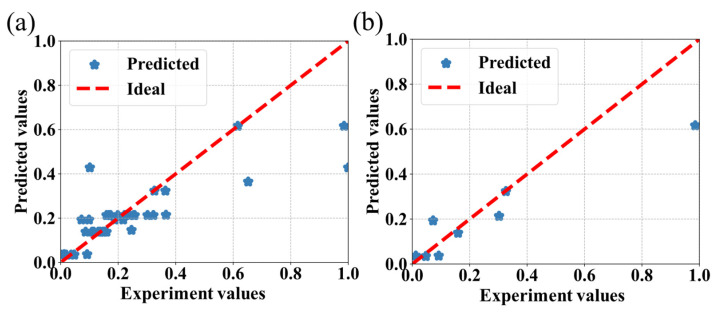
Performance of the AdaBoost algorithm for the prediction of the IBU loading capacity of MOFs associated with the experimental values. (**a**) The predictions are calculated on the entire dataset. (**b**) The predictions are exclusively calculated on the testing dataset.

The prediction results obtained from the CatBoost, SVR, RF, and AdaBoost algorithms are shown in Figure 4, Figure 5, Figure 6 and Figure 7, respectively, where the red line is the ideal prediction line which can visually evaluate the performance of the predictions. It reflects that the results are densely distributed on both sides of the ideal line. Moreover, the analysis of the results of the CatBoost algorithm show that the RMSE is around 9.81%. The R Squared (R^2^) of the testing dataset is 0.76. 

Table 2 shows the R^2^ and RMSE of the different algorithms mentioned above, indicating that the CatBoost algorithm had the best performance among the four algorithms.

The interpretability of the model is becoming an important research trend in machine learning. SHAP “https://shap.readthedocs.io/en/latest/index.html(accessed on 16 June 2022)” originates from a cooperative game theory, where all the features are described as “contributors”. It is also a “model explanation” package that can explain the outputs of any machine learning model References. For instance, the model generates a prediction for each MOF sample of the IBU loading capacity, and the values of SHAP are the contribution index assigned to each feature in the example. Moreover, the greatest strength of the SHAP is that it intuitively reflects the different influent weights of each element in the samples. Then, the features are ranked according to the average absolute value of SHAP, which is the most crucial feature of the model. Meanwhile, this interpretation method is also essential to verify predictions obtained by the model, which are determined by a correct understanding of each feature’s significance.

**Figure 8:**
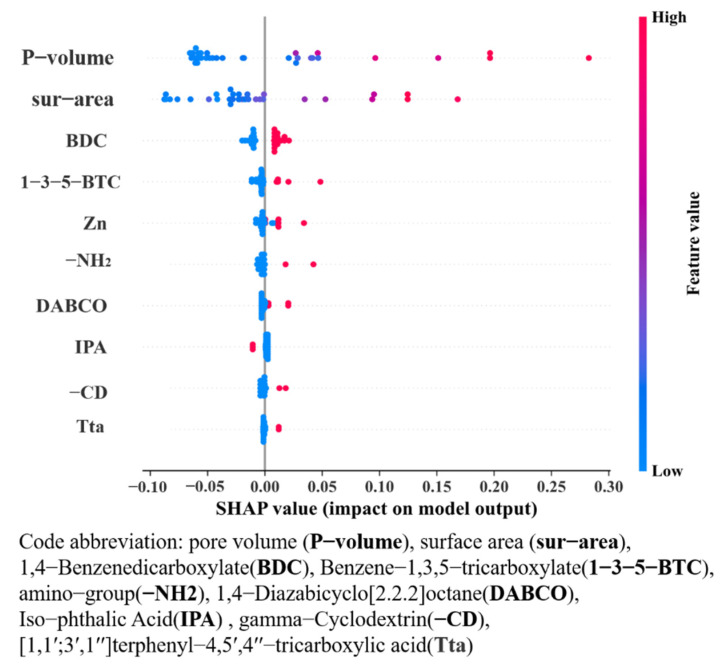
The SHAP plot for the visualization of the main effects of the features.

In this study, the model was well trained to show a satisfactory predictive performance. It offered the SHAP value of the top 10 variables that had the most significant influence on the model predictions, as shown in Figure 8, along with its calculated values in a descending order. In Figure 8, the row represents a feature with the value of SHAP on the bottom horizontal coordinate. We analyzed the distribution of SHAP values for each feature according to different features at the vertical coordinate. The overlapping points fluctuate on the vertical coordinates, and many samples are clustered around the zero centerlines when the features are less important to the model. Additionally, the outstanding features are ranked according to their importance and prioritization, such as specific surface area (sur-area), pore volume (P-volume), 1,4-Benzenedicarboxylate (BDC), etc. More prominently, specific surface area and pore volume influence the model’s outputs more than the other features, based on the SHAP. 

**Figure 9:**
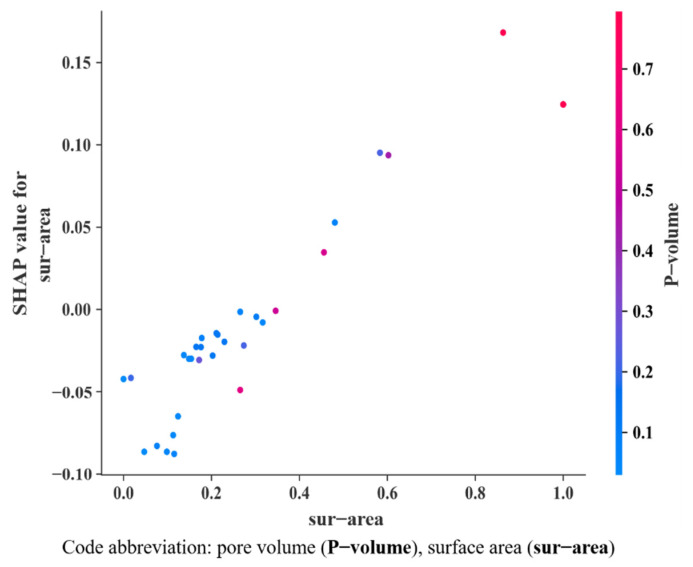
SHAP dependence plot for visualization of the interaction between sur-area and P-volume.

Figure 9 reveals the top two feature combinations of the specific surface area (sur-area) and pore volume (P-volume). The values of SHAP gradually increase along the main diagonal. This indicates that the interaction coefficient between them is positive, and this combined feature effect is similar to the extrapolation of a single feature effect, which means that their effect becomes significant in the model.

Notably, some dots on the bottom are not crucial for most of the dataset. However, they may be necessary for a small fraction of the dataset for the reason that the results in the formation represent the global variables, not the local variables.

### 3.3. Discussion

Herein, we used different ML algorithms to predict the IBU loading capacity of MOFs based on their structural properties parameters. This method is called “supervised” learning in the field of machine learning. Specifically, the SVR, RF, Adaboost, and Catboost algorithms were involved in supervised learning. In “supervised learning”, the algorithm learns from the labeled examples with the known outcome and generalizes predictions on future data where the result is unknown.

Training and comparing different algorithms is critical for developing preferred results and adopting suitable predicted algorithms. We noted that different algorithms had multiple performances on various issues; thus, better-suited algorithms exist for the problems considered in this work. More specifically, the performance estimation revealed that CatBoost had greater superiority in the sparse matrices and could match other advanced machine learning methods among the four algorithms. Its most unique strength is that it processes the categorical features during the training rather than during the features preprocessing stage. In other words, it reduces the need for significant hyperparameter tuning, minimizes the possibility of over-fitting, and makes the model more universal.

In the traditional GBDT algorithm, the most straightforward approach is to replace the categorical features with the average of the corresponding labels [52]. In the decision tree, the average value of the label will be used as the criterion for node splitting [51]. This approach has a disadvantage: features usually contain more information than labels, and if the average of the labels is forced to represent the features, the problem of conditional bias will occur when the data structures of the training and testing dataset are distributed differently [54,55]. Herein, the CatBoost algorithm cannot only solve the above problem, but it can also improve the performance of the model regarding prediction bias.

Finally, the data of MOFs collected from the structure library “http://www.chemsoon.com.cn/ (accessed on 15 May 2022)” was input into the trained model for screening the MOFs with a higher IBU loading capacity. Meanwhile, the predictions of IBU loading capacity are obtained according to this approach, and the results are shown in Table 3. As shown in the last column of the table, the results indicate that the IBU loading capacity in the collected data is in the range of 0.31–0.54 (g/g). Among these, MIL-101(Cr) has the highest IBU loading capacity, and we also found that its pore volume and surface area were the most prominent for the drug loading capacity. Then, we combined the conclusions from the SHAP value that pore volume and surface area have the greatest effect on the predictions. Therefore, this step verifies the reliability of our method of using ML to predict the IBU loading capacity and provides ideas for other bio-nanomaterial studies.

In the future, we can collect more data and develop our own database. In the database, we will utilize ML algorithms to train more MOFs on structural data. Then, we can predict additional physicochemical properties. Furthermore, the model’s performance is expected to further improve if more datasets of MOFs are included, which is consistent with the properties of machine learning. At the same time, we also hope that these predictions will be experimentally validated in future studies. 

## 4. Conclusions

In this study, we have demonstrated that the Catboost algorithm, incorporating both training and testing data, could serve as an efficient preliminary tool for predicting the IBU loading capacity of MOFs. The good performance of the model suggests that the prediction of the screening of MOFs with high IBU loading capacity has been obtained and evaluated, with the expected effects. Meanwhile, the combined effects between two features were visualized by SHAP dependence plots. The results reveal the strength of feature interactions in the used dataset, which are essential in the IBU loading capacity of MOFs. Moreover, we used the AL algorithm to fill the gap in machine learning to predict the IBU loading capacity of MOFs. The improvement in the model also provides a valuable reference for predicting other MOFs’ structural properties. In future studies, we can include more datasets or databases and access even better ensemble algorithms and deep learning networks. We will use these AI methods to fully train the physicochemical properties of bionanomaterials to predict their structures more widely and accurately. With these efforts, it is believed that additional researchers can cooperate more effectively to push for the next frontier of AI combined with the structure of MOFs, accelerating further biomaterial development.

## Figures and Tables

**Table 1:** Sample of partial data and the predicted target (IBU loading capacity) list.

MOFs	Metal Ions	Organic Linkers	Surface Area m^2^/g	Pore Volume cm^3^/g	IBU Loading Capacity g/g	Reference
MIL-100	Cr	BDC	3340	1.160	0.350	[16]
MIL-101	Cr	BDC	5510	2.020	0.140	[16]
MIL-53(Cr)	Cr	BDC	1500	1.600	0.220	[40]
UMCM-1	Zn	BDC, BTC	4764	2.280	1.360	[41]
MIL-100(Fe)	Fe	BDC	1900	0.590	0.330	[42]
[Zn(BDC)(H2O)2]n	Zn	BDC, DABCO	1545	0.669	0.445	[43]
MIL-53	Fe	BDC	954	0.479	0.231	[44]
…	…	…	...	...	...	…
CD-MOF-1	K	γ-CD	1220	0.493	0.274	[44]
MIL-47	V	BDC	729	0.270	0.120	[45]
MIL-53	Cr	BDC	864	0.290	0.190	[45]

Abbreviations: 1,4-Benzenedicarboxylate (BDC), Benzene-1,3,5-tricarboxylate (BTC), 1,4-Diazabicyclo [2.2.2]octane (DABCO), gamma-Cyclodextrin (γ-CD).

**Table 2:** Comparison of R^2^ and RMSE of different algorithms.

Algorithm	R^2^	RMSE (%)
AdaBoost	0.66	12.10
SVR	0.70	10.53
RF	0.72	9.62
CatBoost	0.76	9.81

**Table 3:** The predictions of IBU loading capacity are based on the improved model and the structural library.

MOFs	Metal Ions	Organic Linkers	The Predictions of IBU Loading Capacity (g/g)
NH2-MIL-101(Fe)	Fe	BDC	0.4999
UIO-66-F4	Zr	BDC	0.3091
UIO-66-(SH)2	Zr	BDC	0.3176
NO2-UIO-66	Zr	BDC	0.3361
MOF-74(Ni)	Ni	BDC	0.3160
NH2-MIL-101(Cr)	Cr	BDC	0.4965
MIL-101(Cr)	Cr	BDC	0.5408
UIO-66	Ni	BDC	0.3285
NH2-UIO-66	Ni	BDC	0.3197

Abbreviation: 1,4-Benzenedicarboxylate (BDC).

## Data Availability

Datasets and original images are available from the corresponding author on request, or can be downloaded in the Supplementary Materials.

## Supplementary Materials

The following supporting information can be downloaded at: https://www.mdpi.com/article/10.3390/bioengineering9100517/s1, Table S1: Hyperparameter and optimal value for each machine ML model; Table S2: Sample of partial data and the predicted target (IBU loading capacity) list.
