# Supplementary material for: Prediction of the Ibuprofen Loading Capacity of MOFs by Machine Learning
Source: Bioengineering (Basel). 2022 Sep 30;9(10):517. doi: 10.3390/bioengineering9100517 (PMC9598200; doi:10.3390/bioengineering9100517)
Supplement: Supplementary file 1 [file bioengineering-09-00517-s001.zip › bioengineering-1838678 - Supplemental Material(s1).pdf]

The specified parameters subset of each ML model has been filled into table S1.

Table S1. Hyperparameter and optimal value for each machine ML model.

Algorithms	Hyperparameters	Search range	Optimal value
AdaBoost	base_estimator	default	AdaBoost Regressor
	n_estimators	from 10 to 100, interval=10	50
	learning_rate	From 0.1 to 1, interval=0.1	1
	loss	linear, square, exponential	linear
SVR	kernel	linear, poly, rbf, sigmoid	rbf
	degree	1,2,3,4	3
	gamma	auto, scale	scale
	coef0	0,0.01.0.001	0
	tol	0.01,0.001,0.0001	0.001
	verbose	True, False	False
	shrinking	True, False	True
	cache_size	1.002E+11	200
	epsilon	0.01,0.05,0.1	0.1
	max_iter	10, 50, 100, 200, -1	-1
RF	n_estimators	from10 to 200, interval=10	100
	max_depth	10, 20, 30, 40, None	None
	min_samples_split	2, 3, 4, 5	2
	min_samples_leaf	1, 2, 3, 4	1
	bootstrap	True, False	True
	max_features	auto, sqrt	auto
CatBoost	loss_function	RMSE, Logloss, MAE	RMSE
	iterations	10, 20, 30	20
	learning_rate	0.1, 0.2, 0.3, 0.4	0.2
	max_depth	from10 to 16, interval=1	11
	nan_mode	Forbidden, Min, Max	Min
	eval_metric	True, False	True
